# Associations between Prenatal Physical Activity and Neonatal and Obstetric Outcomes—A Secondary Analysis of the Cluster-Randomized GeliS Trial

**DOI:** 10.3390/jcm8101735

**Published:** 2019-10-19

**Authors:** Julia Hoffmann, Julia Günther, Kristina Geyer, Lynne Stecher, Julia Kunath, Dorothy Meyer, Monika Spies, Eva Rosenfeld, Luzia Kick, Kathrin Rauh, Hans Hauner

**Affiliations:** 1 Else Kröner-Fresenius-Centre for Nutritional Medicine, Klinikum rechts der Isar, Technical University of Munich, Georg-Brauchle-Ring 62, 80992 Munich, Germany; 2 Competence Centre for Nutrition (KErn), Am Gereuth 4, 85354 Freising, Germany

**Keywords:** physical activity, exercise, lifestyle intervention, pregnancy, neonatal outcomes, obstetric outcomes, obesity prevention, routine care, birth weight, large for gestational age

## Abstract

Prenatal physical activity (PA) was discussed to decrease the incidence of obstetric and neonatal complications. In this secondary cohort analysis of the cluster-randomized GeliS (“healthy living in pregnancy”) trial, associations between prenatal PA and such outcomes were investigated. PA behavior was assessed twice, before or during the 12th week (baseline, T0) and after the 29th week of gestation (T1), using the self-reported Pregnancy Physical Activity Questionnaire. Obstetric and neonatal data were collected in the routine care setting. Data were available for 87.2% (*n* = 1994/2286) of participants. Significant differences between the offspring of women who adhered to PA recommendations at T1 and offspring of inactive women were found in birth weight (*p* = 0.030) but not in other anthropometric parameters. Sedentary behavior was inversely associated with birth weight at T1 (*p* = 0.026) and, at both time points, with an increase in the odds of low birth weight (T0: *p* = 0.004, T1: *p* = 0.005). Light-intensity PA at T0 marginally increased the odds of caesarean section (*p* = 0.032), but neither moderate-intensity nor vigorous-intensity activity modified the risk for caesarean delivery at any time point. The present analyses demonstrated associations between prenatal PA and some neonatal and obstetric outcomes.

## 1. Introduction

Worldwide, more than 41 million children under 5 years of age are affected by childhood obesity and being overweight, which poses one of the major challenges of the 21st century [1]. Research suggests that babies born with specific anthropometric characteristics are more susceptible to childhood obesity and to an increased risk of obesity later in life [2]. For example, neonatal outcomes such as high birth weight (>4000 g) and being born large for gestational age (LGA; >90th percentile for gestational age) were shown to be early markers for an increased obesity risk in infancy [3,4].

A healthy lifestyle during pregnancy is discussed to enhance the maternal health status, positively impact fetal development, and improve neonatal as well as obstetric outcomes [5,6]. Within the last few years, approaches focusing on the prenatal lifestyle of the mother-to-be have been initiated to avoid an early obesogenic environment in utero. Lifestyle interventions frequently target a modification of the maternal diet and/or physical activity (PA) behavior [7]. However, the role of PA in the prevention of adverse neonatal and obstetric complications is controversially discussed. While some reported PA to lower the risk for LGA and high birth weight [8,9], others suggested positive associations between PA and birth weight dependent on the PA intensity [10]. Moreover, the effect of prenatal PA on the risk for preterm and caesarean delivery is highly debated, as some analyses showed beneficial associations between prenatal PA and the risk of preterm birth and cesarean section, while others did not demonstrate any effects of prenatal PA on gestational age at birth or mode of delivery [11,12,13,14,15]. Thus, evidence is still inconclusive. The activity mode, time point during pregnancy, and intensity seem to be particularly important in this heterogeneous picture on the effect of PA on neonatal and obstetric parameters. Nevertheless, no negative effects of prenatal PA on obstetric or neonatal outcomes have been found so far.

In order to encourage expectant mothers to engage in adequate levels and intensity of PA, the American College of Obstetricians and Gynecologists (ACOG) established recommendations in the report “Physical Activity and Exercise During Pregnancy and the Postpartum Period” [16]. These recommendations have been adapted for Germany [17]. The key message of these recommendations is that pregnant women without contraindications should not stop exercising during the course of pregnancy. In the absence of medical or obstetric complications, pregnant women are recommended to engage in moderate-intensity PA for at least 20–30 min per day on most or all days of the week [16,17].

In Bavaria, Germany, the large-scale “Gesund leben in der Schwangerschaft”/ “Healthy living in pregnancy” (GeliS) trial was initiated offering a lifestyle intervention program that is linked to routine prenatal care [18]. The GeliS trial sought to improve maternal and neonatal health outcomes as well as obstetric parameters in order to positively influence the long-term health development of both mothers and infants. The primary objective of the GeliS trial was to reduce the proportion of women with excessive gestational weight gain. The GeliS intervention was not successful in reducing the incidence of excessive gestational weight gain [19]. However, it led to some improvements in maternal antenatal dietary [20], and PA behavior [21]. Moreover, some minor but likely clinically irrelevant benefits in maternal postpartum weight development and breastfeeding behavior were observed [22].

The present work aims to investigate associations between early and late prenatal PA behavior and neonatal and obstetric parameters in the entire GeliS cohort. Moreover, we sought to describe differences in neonatal and obstetric outcomes between women meeting or not meeting the PA recommendations given by the ACOG.

## 2. Materials and Methods

### 2.1. The GeliS Study

The GeliS study is a prospective, multicenter, cluster-randomized, controlled, open intervention trial that was conducted alongside routine prenatal care with the aim to improve prenatal weight development and reduce the risk for adverse maternal and infant health outcomes. A detailed description of the study design, setting, population, and randomization has already been published elsewhere [18]. In brief, between 2013 and 2015, women with (1) a pre-pregnancy body mass index (BMI) between ≥ 18.5 kg/m^2^ and ≤ 40.0 kg/m^2^, (2) a singleton pregnancy, (3) age between 18 and 43 years, (4) sufficient German language skills, and (5) stage of pregnancy before the end of the 12th week of gestation were recruited by gynecological and midwifery practices in five administrative regions of Bavaria depicting the “real-life” setting of routine prenatal care. Written informed consent for participation was given by all participants.

Apart from routine prenatal care, participants of the control group (C) obtained only general information on a healthy antenatal lifestyle via a flyer. Participants of the intervention group (IV) additionally received a comprehensive lifestyle intervention program consisting of three antenatal counselling sessions and one postpartum face-to-face counselling session given by previously trained midwives, medical personnel, or gynecologists. Within these sessions, the importance of a healthy prenatal and postnatal lifestyle was addressed and practice-oriented advice was given in accordance with national and international recommendations [16,17]. A detailed description of the counselling content has already been reported [18].

The study was performed in accordance with the current local regulatory requirements and laws as well as with the declaration of Helsinki. The Ethics Commission of the Technical University of Munich approved the study protocol. The trial is registered at the ClinicalTrials.gov Protocol Registration System (NCT01958307) [23].

The intervention yielded some improvements in the PA behavior [21] but no major between-group differences in primary and secondary maternal, neonatal, and obstetric outcomes except for infants’ birth weight and height [19]. Therefore, we made the post-hoc decision to pool data from the IV and C for the present analysis and adjusted for the group assignment.

### 2.2. Data Collection and Outcomes

At the time of recruitment (before the end of the 12th week of gestation), all baseline characteristics were collected through a screening questionnaire. Pre-pregnancy BMI was calculated based on self-reported weight. Infant anthropometrics, including neonatal and obstetric parameters, were retrieved from maternity and birth records. Preterm birth was defined as delivery before the 37th week of gestation. Newborn’s birth weight below 2500 g was described as “low birth weight,” above 4000 g as “high birth weight” and above 4500 g as “macrosomic.” Offspring whose weight was above the 90th percentile for gestational age was defined as “large for gestational age” (LGA) and whose weight was below the 10th percentile for gestational age as “small for gestational age” (SGA). BMI-z-scores were calculated on the basis of the German reference group, according to Kromeyer-Hauschild et al. (2001) [24].

Prenatal PA behavior was assessed twice, at T0 (before or in the 12th week of gestation) and at T1 (after the 29th week of gestation), using the validated Pregnancy Physical Activity Questionnaire (PPAQ) [25], which was slightly adapted to German habits. Data were self-reported without supervision. In the PPAQ, participants were asked to estimate the mean time spent engaging in 32 activities in the past month. In two included open-ended questions, participants were able to name activities that were not specifically listed in the PPAQ.

For the analyses of the PPAQ data, we made use of the widely applied concept of a metabolic equivalent of task (MET), which is a procedure to express energy costs of PA as multiples of the resting metabolic rate [26,27]. One MET equals the resting metabolic rate during sitting [26]. The 2011 Compendium of Physical Activities lists MET values for different types of sports and, thus, activity-specific multiples of the resting metabolic rate [28]. Moreover, the PPAQ evaluation instruction indicates MET values for activities that are assessed in the PPAQ [29].

PPAQ data were processed to receive a measure of average weekly energy expenditure in MET-h/week by multiplying the number of hours spent in each activity by its corresponding intensity (MET). Corresponding MET values were retrieved from the evaluation instruction of the PPAQ [29]. Using the 2011 Compendium of Physical Activities [28], the corresponding MET values were assigned to reported activities in open-ended questions. As described by the authors [29], calculated average weekly energy expenditure was grouped into activity “types” and activity “intensities” or summed up into “total PA” and “total PA of light intensity and above” (TALIA). Types included the categories “household/caregiving”, “transportation”, and “occupational activities”, as well as “sport/exercise” and “inactivity”. Intensities were sub-grouped into “sedentary” (MET < 1.5), “light” (MET ≥ 1.5 and < 3.0), “moderate” (MET ≥ 3.0 and ≤ 6.0), or “vigorous” activities (MET > 6.0). As done by others [7], questionnaires were excluded from the analysis due to over-reporting if the total number of reported hours exceeded the total number of hours per week. Moreover, if participants indicated to have spent more than 12 hours per day for seven days per week in occupational activity, they were defined as an over-reporter for this specific category and not considered in “occupational activity” analyses. To obtain a dichotomized variable indicating whether women met the national and international PA recommendations [16,17], the threshold of ≥ 7.5 MET-h/week in sport activities of moderate intensity or above was used. This procedure was recommended by the PPAQ developer (personal communication) and was applied by others [30]. Women with a level of ≥ 7.5 MET-h/week in sport activities of moderate-intensity or above, thus, meeting the recommendations at one time point (T0 or T1), were defined as “active”, whereas the “inactive” group was characterized by values below this threshold at one time point. In sub-analyses, women meeting the recommendations at both time points were named “active^T0+T1^” and women meeting the recommendations never or only at one time point were described as “inactive^T0+T1^”.

### 2.3. Statistical Analysis

Power calculation was based on the primary study outcome and has been described elsewhere [18]. All presented analyses were performed using SPSS software (IBM SPSS Statistics for Windows, version 24.0, IBM Corp, Armonk, NY, USA). The subsequent analyses included all participants except for the ones that either dropped out before delivery due to miscarriage or late loss of pregnancy, terminations, pregnancy complications interfering with the intervention, and maternal deaths or the ones that did not provide any of the three infant parameters: birth weight, birth length, and head circumference. Moreover, participants were excluded from the analysis of single PA intensities or types if one corresponding question was not answered.

Baseline characteristics, infant anthropometrics, obstetric, and neonatal outcomes are presented as proportions or as mean ± standard deviation (SD) if appropriate. PA behavior of active and inactive participants is characterized by type and intensity of PA and is presented in mean MET-h/week ± SD. To assess differences in anthropometric, neonatal, and obstetric outcomes between active and inactive or active^T0+T1^ and inactive^T0+T1^ women, respectively, general linear regression models were applied for continuous variables and binary logistic regression models for dichotomized outcomes. Thereby, unadjusted as well as adjusted models were performed controlling for the pre-pregnancy BMI category, parity, age, and group assignment. Similarly, linear and binary logistic regression models were used to explore potential associations of a change in PA intensities and TALIA by 10 MET-h/week with obstetric and neonatal outcomes adjusting for the same confounding factors. *p*-values below 0.05 were considered to be statistically significant.

## 3. Results

### 3.1. Participant Flow and Baseline Characteristics

**Figure 1:**
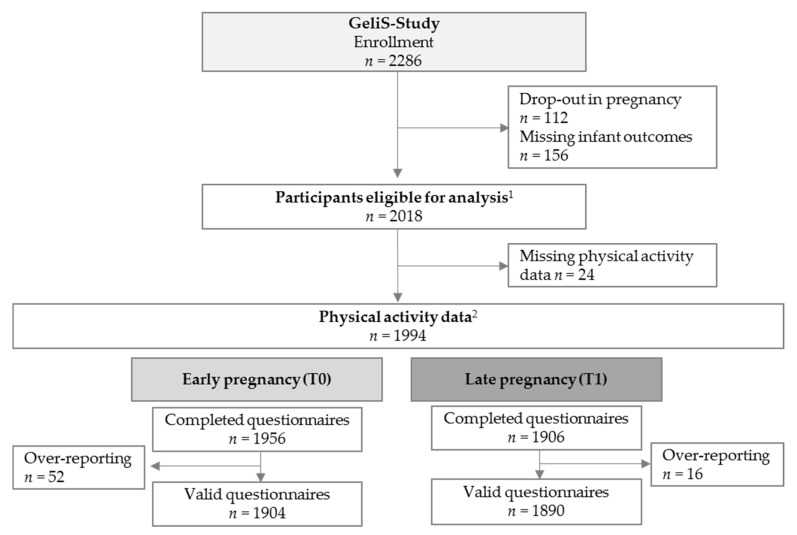
Participant flow and availability of physical activity data. ^1^ Women without miscarriages, late loss of pregnancy, terminations, pregnancy complications that interfere with the intervention, maternal deaths, and/or that provided at least one of the three infant outcomes (birth weight, birth length, and head circumference). ^2^ Women who provided PA data at T0 or T1. Abbreviations: PA: physical activity, T0: assessment before or in the 12th week of gestation, and T1: assessment after the 29th week of gestation.

A total of 2286 women were enrolled in the GeliS study (Figure 1). Among them, *n* = 112 participants dropped out during the course of pregnancy and, for *n* = 156 participants, the minimum of infant outcomes was not available. Thus, 2018 women were eligible for analysis of whom *n* = 24 provided neither PA data at T0 nor at T1, resulting in *n* = 1994 women with PA data at either one or both time points. After the exclusion of over-reporters, *n* = 1904 valid questionnaires were available at T0 and *n* = 1890 questionnaires at T1.

Maternal characteristics, obstetric outcomes, and corresponding neonatal parameters of all eligible subjects are depicted in Table 1.

### 3.2. Associations Between Prenatal Physical Activity and Infant Anthropometrics, Neonatal, and Obstetric Outcomes

Among all women providing valid PA data, 47.0% met the PA recommendations at T0 and 56.2% at T1. Among participants providing valid data for both time points, 597 (33.1%) women met the recommendations at both time points. Obstetric parameters as well as corresponding anthropometrics and neonatal outcomes of offspring of women considered active and inactive at T0 and T1 are shown in Table 2. Results of unadjusted analyses are shown in Table S1. There was evidence that infants of women who were active in late pregnancy (T1) had a higher birth weight (3364.5 ± 481.0 g) than infants whose mothers were inactive at T1 (3341.4 ± 492.5 g, adjusted effect size 49.74, 95% CI 4.94 to 94.53, *p* = 0.030). No significant differences in other anthropometric parameters such as length, BMI, head circumference, or BMI-z-score were detected either at T0 or at T1. In the adjusted analysis, preterm birth was less likely among women who were active at T1 compared to women who were inactive (adjusted OR 0.66, 95% CI 0.44 to 0.98, *p* = 0.038).

The odds of LGA tended to be higher at both time points for offspring of women adhering to the PA recommendations (T0: adjusted OR 1.37, 95% CI 0.96 to 1.94, *p* = 0.079, T1: adjusted OR 1.39, 95% CI 0.97 to 2.00, *p* = 0.075), but statistical evidence was lacking. No significant differences between active and inactive women were observed in other neonatal and obstetric outcomes at any time, neither in the unadjusted (Table S1) nor in the adjusted analysis (Table 2).

We found evidence that infants of women who adhered to PA recommendations at both time points (active^T0+T1^) were more likely of being born LGA when compared to infants of women who either met the recommendations once or at no time point (Table S2, adjusted *p* = 0.025). Moreover, these infants showed, by trend, an increased risk for high birth weight (Table S2, adjusted *p* = 0.095), but statistical evidence was lacking.

Table 3 shows the effect of different PA intensities on infant anthropometrics as well as obstetric outcomes and Table 4 on neonatal outcomes. Corresponding unadjusted models are presented in the supplement (Table S3 and Table S4). The level of sedentary PA in late pregnancy was significantly and inversely associated with infant birth weight (Table 3, adjusted *p* = 0.026) and positively associated with the odds of low birth weight at both time points (Table 4, T0: adjusted *p* = 0.004, T1: adjusted *p* = 0.005). Moreover, the level of sedentary-intensity was, by trend, associated with increasing odds of preterm birth at T0 and T1 (Table 3, T0: adjusted *p* = 0.051, T1: adjusted *p* = 0.070) like vigorous activity at T1 (Table 3, adjusted *p* = 0.081), but there was no statistically significant evidence for these findings. The level of TALIA and light-intensity activities at T0 was related to a marginal increase in the odds of caesarean section (Table 3, TALIA: adjusted *p* = 0.040, light-intensity PA: adjusted *p* = 0.032).

The level of moderate-intensity activity at both time points was, by trend, positively associated with the odds of high birth weight (Table 4, T0: adjusted *p* = 0.080, T1: adjusted *p* = 0.050). The level of light-intensity activities at T0 seemed to be linked to decreasing odds of SGA (Table 4, adjusted *p* = 0.053). However, none of these trends were statistically significant.

## 4. Discussion

In this secondary analysis of the GeliS cohort, we were able to show associations between early and late prenatal PA and infant anthropometrics as well as several neonatal and obstetric outcomes. Moreover, we could comprehensively investigate the influence of differences in the PA behavior between women who met and did not meet the prenatal PA recommendations on these outcomes. Lastly, we could associate different PA intensities with infant anthropometrics and the risk of adverse neonatal and obstetric outcomes.

Infants of women meeting the PA recommendations in late pregnancy were born significantly heavier and tended to be larger than offspring of inactive women. However, the estimated differences between groups were small. We observed that late sedentary PA was inversely associated with birth weight and positively associated with the risk of low birth weight but not for SGA. Engaging in light-intensity activities in early pregnancy was associated with a decreasing risk of the offspring to be born SGA. Our findings on infant birth weight correspond to the results of Koushkie Jahromi et al. [31] and Badon et al. [32] who, likewise, reported that babies born to women who exercised during pregnancy were heavier than those born to non-exercising women [31] with no impact of early sedentary behavior on birth weight [32]. However, our results contrast the observations of Bisson et al. [33] who also used the PPAQ to explore effects of PA on infant birth weight in a Canadian cohort. Although study samples seemed to be comparable in terms of BMI, educational level, and parity, these authors observed a reduction in infant birth weight by 2.5 g with each increase of 1 MET-h/week in the level of sports and exercise [33]. A possible explanation for the discrepancy with our results might be that the authors included more covariates in their model such as maternal education and smoking status, paternal weight, and infant sex, among others.

In general, the opinion about the effect of prenatal PA on infant birth weight is conflicting. Some studies report no significant association [14,34,35,36], while others report a positive [31,37] or a negative association [38,39]. The considerable heterogeneity in the assessment of PA data complicates the comparison of study results. Furthermore, inconsistency of results may arise from differences in type, frequency, timing, and duration of PA [40]. There is some evidence suggesting an inverted U-shaped relationship between PA intensity and birth weight [10]. Although this hypothesis has been criticized [9], it provides a possible explanation for our observations and seems to explain why we found late sedentary-intensity to be inversely associated with birth weight and positively associated with the risk for a low birth weight, while moderate-intensity PA in early and late pregnancy appeared to be linked to a slight increase in the odds of high birth weight. Bisson et al. [10] explain this relationship by alterations in glucose availability and uteroplacental blood flow in response to exercise [41], which seems to be reduced by exercise intensity [42,43] and compensated afterward, resulting in enhanced fetal growth. A comprehensive estimation of the influence of PA on neonatal body composition might elucidate the debate on its effect on birth weight [35].

In our cohort, infants of active women at T0 and T1 tended to be at higher risk for being born LGA. This observation was shown to be significant in women meeting the recommendations in early as well as in late pregnancy. However, it is difficult to explain this finding since we could not identify any PA intensity during the course of pregnancy, which was associated with an increasing risk of LGA. Current research has either found no influence of PA on the incidence of LGA [12], or observed PA to be protective against LGA [8,40,44], which calls into question whether our observations were chance findings.

Our results showed that women who were active in late pregnancy were less likely to give birth prematurely, which was similarly observed by others [45]. Likewise, we identified sedentary PA in early and late pregnancy to be, by trend, associated with an increase in the odds of preterm delivery, even though statistical evidence was lacking. These observations are in line with some investigations showing either no effect of PA on the risk for preterm birth [12,13,46], or a protective influence [47]. However, we observed that vigorous-intensity PA in late pregnancy appeared to be linked to an increasing risk for preterm birth. This is in contrast with observations from a prospective study [48], a systematic review [49], and a meta-analysis [11], which showed that higher leisure-time and vigorous PA did not change the incidence of, or reduce the risk for preterm deliveries. Nevertheless, the impact of prenatal PA on pregnancy duration was studied in women with different weight classifications, which might explain inconsistencies with our results since not all trials included women with normal and overweight outcomes simultaneously.

In the present analyses, we found no difference in the incidence of caesarean delivery between active and inactive women during the course of pregnancy. This corresponds to other current investigations [13,36,50] even though some studies observed a lower caesarean section rate in exercising women [8,14,46]. However, we observed that TALIA and light-intensity PA in early pregnancy were associated with a marginal increase in the odds of caesarean delivery. Poyatos-Léon et al. [15], likewise, assessed the association between PA intensities and the risk for caesarean section deliveries and provided a possible extension of our findings. They concluded that, in general, exercise during pregnancy appears to decrease the risk of caesarean delivery. In particular, women who engaged in exercise during the second and third trimester seemed to increase their likelihood of a normal delivery [15].

This secondary analysis of the GeliS trial has some limitations. As noted by others [40], we did not include dietary intake as a covariate. Previously, we reported small effects of the maternal diet on neonatal weight-related parameters [51], and are aware that maternal dietary behavior might have biased the present results. Further potential confounders such as the maternal employment, living circumstances, or other lifestyle factors (smoking, drinking) might have influenced the maternal PA level, but were not considered in the present analyses. Moreover, we used a self-administered PPAQ, which, although a being validated and easily applicable tool, was filled out by participants at both time points without supervision or accompanying interview. Self-reports rely on the subjective estimation of participants who had to remember their PA level and type of performed sports for the past four weeks. As reported by others [52], the presented self-reported data might have been susceptible to over-reporting and under-reporting. Despite our efforts to exclude over-reporting, this mode of PPAQ self-administration might have biased our results. While done by others [30] and recommended by the questionnaire developers (personal communication), assessing the adherence to the PA recommendations by means of the PPAQ might present a methodological shortcoming and might explain some inaccuracies with observed effects of PA intensities. We acknowledge that objectively measuring prenatal PA using accelerometers might provide a more precise assessment and might provide a more accurate estimation of changes and variations in the PA level during the course of pregnancy. Since the GeliS trial was performed in a large cohort within the real-life setting of routine prenatal care, using other methods of PA data collection in place of or in addition to the PPAQ was not feasible. A minor limitation is that our cohort differed slightly from the general German population of women of child-bearing age in terms of educational level and BMI categories [53], which needs to be considered when generalizing our findings. Moreover, neonatal and obstetric outcomes were collected from different hospitals, and data collection was, thus, not completely standardized. In addition, there was no possibility to collect offspring´s body composition measurements, which may have expanded some of our findings beyond associations with crude measurements of body weight and BMI. Lastly, we are aware that some pregnancy-induced complications such as premature contractions that potentially led to a reduction of maternal PA, could have resulted in obstetric complications (e.g., preterm birth) and could have biased our results.

There are several strengths of this analysis that merit particular attention. Current research has either investigated women with normal weight or studied women with overweight and obesity. The GeliS cohort comprised women of all BMI categories, which allowed us to report the impact of prenatal PA for women in all weight ranges. Irrespective of shortcomings, the PPAQ is a valid and easily applicable tool that enables an extensive description of PA behavior during pregnancy. Using the PPAQ, we could not only estimate the impact of different PA intensities, but could also sub-group participants according to their activity level into meeting or not meeting the ACOG prenatal PA recommendations. This gave us the opportunity to report differences in obstetric and neonatal outcomes between sub-groups. Data for this study were collected within a public health approach under real-life conditions without requiring further measurements or tools, which allowed for methodological advantages. First, we were able to follow participants and collect data longitudinally over the course of pregnancy, which allowed us to observe the impact of prenatal PA in early as well as in late pregnancy. Second, we were able to collect data on a relatively large study sample. This provided a comprehensive and valuable assessment of the influence of antenatal PA on infant health outcomes as well as obstetric parameters.

To the best of our knowledge, there is no other trial that has provided a comprehensive description of the impact of early as well as late prenatal PA on obstetric and neonatal outcomes in such a large sample and that was additionally able to demonstrate the influence of PA intensities on these parameters.

## 5. Conclusions

This secondary analysis of the large-scale GeliS cohort demonstrated moderate differences in offspring anthropometrics and obstetric as well as neonatal outcomes associated with maternal PA behavior. Moreover, the odds of adverse neonatal and obstetric outcomes seemed to be dependent on the intensity and timing of prenatal PA. Future research should concentrate on offspring´s body composition to expand current investigations and to provide more insight into the clinical meaning of such findings. In terms of health benefits for the mothers-to-be and their offspring, it remains a challenge to characterize the optimal intensity level of antenatal PA. A follow-up of infants may help to reveal the long-term impact of PA during pregnancy on infant health and its potential contribution in the development of childhood obesity.

## Figures and Tables

**Table 1:** Maternal, neonatal, and obstetric characteristics of eligible participants.

Characteristics	Total (*n* = 2018)
**Maternal Characteristics**	
Pre-pregnancy age, years	30.3 ± 4.4
Pre-pregnancy weight, kg	68.2 ± 13.4
Pre-pregnancy BMI, kg/m^2^	24.4 ± 4.5
**Pre-Pregnancy BMI Category**	
BMI 18.5–24.9 kg/m^2^	1311/2018 (65.0%)
BMI 25.0–29.9 kg/m^2^	464/2018 (23.0%)
BMI 30.0–40.0 kg/m^2^	243/2018 (12.0%)
**Educational Level**	
General secondary school	320/2014 (15.9%)
Intermediate secondary school	856/2014 (42.5%)
(Technical) High school	838/2014 (41.6%)
**Country of Birth**	
Germany	1790/2014 (88.9%)
Others	224/2014 (11.1%)
**Nulliparous**	1162/2018 (57.6%)
Living with a partner	1939/2011 (96.4%)
Full-time employed	1056/1996 (52.9%)
**Neonatal and Obstetric Characteristics**	
Birth weight, g	3337.6 ± 517.8
Birth length, cm	51.3 ± 2.6
Head circumference, cm	34.7 ± 1.6
BMI, kg/m^2^	12.7 ± 1.3
BMI-z-Score ^a^	0.04 ± 1.02
LGA	148/2016 (7.3%)
SGA	172/2016 (8.5%)
Low birth weight	101/2018 (5.0%)
High birth weight	169/2018 (8.4%)
Macrosomia	19/2018 (0.9%)
Preterm birth	132/2016 (6.5%)
Caesarean section	582/2017 (28.9%)

Depicted are mean ± SD or proportions (percent). ^a^ BMI-z-score was calculated using German standards [24]. Abbreviations: BMI: body mass index, LGA: large for gestational age, and SGA: small for gestational age.

**Table 2:** Differences between active and inactive women in infant anthropometrics, neonatal, and obstetric outcomes.

	Time Point	Active		Inactive			
**Anthropometrics**		***n*^a^**	**Mean** **±** **SD**	***n*^a^**	**Mean** **±** **SD**	**Adjusted Effect Size ^b^ (95% CI)**	**Adjusted *p* Value ^b^**
**Birth weight, g**	T0	*n* = 893	3338.0 ± 527.9	*n* = 1008	3337.4 ± 508.9	11.44 (−35.02, 57.91)	0.629
	T1	*n* = 1061	3364.5 ± 481.0	*n* = 827	3341.4 ± 492.5	49.74 (4.94, 94.53)	0.030
**Birth length, cm**	T0	*n* = 885	51.3 ± 2.6	*n* = 1000	51.4 ± 2.6	−0.00 (−0.24, 0.23)	0.980
	T1	*n* = 1056	51.4 ± 2.4	*n* = 824	51.3 ± 2.6	0.23 (−0.00, 0.46)	0.054
**Head circum-ference, cm**	T0	*n* = 875	34.7 ± 1.6	*n* = 989	34.7 ± 1.6	−0.02 (−0.17, 0.12)	0.762
	T1	*n* = 1047	34.8 ± 1.5	*n* = 814	34.7 ± 1.6	0.11 (−0.04, 0.25)	0.148
**BMI, kg/m^2^**	T0	*n* = 885	12.7 ± 1.3	*n* = 1000	12.6 ± 1.2	0.04 (−0.08, 0.15)	0.503
	T1	*n* = 1056	12.7 ± 1.3	*n* = 824	12.7 ± 1.2	0.09 (−0.03, 0.20)	0.140
**BMI-z-score^c^**	T0	*n* = 884	0.05 ± 1.07	*n* = 1000	0.03 ± 0.98	0.04 (−0.06, 0.13)	0.463
	T1	*n* = 1055	0.07 ± 1.04	*n* = 824	0.04 ± 0.96	0.07 (−0.02, 0.16)	0.134
**Neonatal and Obstetric Outcomes**			*n* (%)		*n* (%)	Adjusted OR ^b^ (95% CI)	**Adjusted *p* Value ^b^**
**LGA**	T0	*n* = 893	74 (8.3)	*n* = 1006	66 (6.6)	1.37 (0.96, 1.94)	0.079
	T1	*n* = 1061	87 (8.2)	*n* = 826	54 (6.5)	1.39 (0.97, 2.00)	0.075
**SGA**	T0	*n* = 893	72 (8.1)	*n* = 1006	92 (9.1)	0.84 (0.61, 1.16)	0.293
	T1	*n* = 1061	100 (9.4)	*n* = 826	61 (7.4)	1.16 (0.82, 1.63)	0.408
**Low birth weight**	T0	*n* = 893	49 (5.5)	*n* = 1008	46 (4.6)	1.16 (0.77, 1.76)	0.485
	T1	*n* = 1061	46 (4.3)	*n* = 827	34 (4.1)	0.95 (0.60, 1.51)	0.835
**High birth weight**	T0	*n* = 893	81 (9.1)	*n* = 1008	80 (7.9)	1.20 (0.86, 1.66)	0.282
	T1	*n* = 1061	92 (8.7)	*n* = 827	66 (8.0)	1.16 (0.82, 1.62)	0.402
**Macrosomia**	T0	*n* = 893	10 (1.1)	*n* = 1008	8 (0.8)	1.37 (0.54, 3.53)	0.509
	T1	*n* = 1061	11 (1.0)	*n* = 827	7 (0.8)	1.18 (0.44, 3.14)	0.746
**Preterm birth**	T0	*n* = 893	61 (6.8)	*n* = 1006	62 (6.2)	1.09 (0.75, 1.57)	0.649
	T1	*n* = 1061	52 (4.9)	*n* = 826	57 (6.9)	0.66 (0.44, 0.98)	0.038
**Caesarean section**	T0	*n* = 893	264 (29.6)	*n* = 1008	278 (27.6)	1.11 (0.90, 1.36)	0.323
	T1	*n* = 1060	301 (28.4)	*n* = 827	232 (28.1)	0.98 (0.80, 1.21)	0.868

Depicted are mean ± SD and proportions (*n* (%)).^a^ Number of participants depend on the availability of anthropometric, neonatal, and obstetric data. ^b^ adjusted for pre-pregnancy age, pre-pregnancy BMI, parity, group assignment. ^c^ BMI-z-score was calculated using German standards [24]. *Active*: Women meeting physical activity recommendations defined as ≥ 7.5 MET-h/week in category sports activity of moderate-intensity or greater. *Inactive*: Women not meeting physical activity recommendations (< 7.5 MET-h/week in category sports activity of moderate-intensity or greater). Abbreviations: BMI: body mass index, LGA: large for gestational age, OR: odds ratio, SGA: small for gestational age, T0: assessment before or in the 12th week of gestation, and T1: assessment after the 29th week of gestation.

**Table 3:** Associations between physical activity intensity and infant anthropometrics and obstetric outcomes.

	Birth Weight		BMI		Preterm Birth		Caesarean Section	
	Adjusted Effect Size ^a^ (95% CI)	Adjusted *p* Value ^a^	Adjusted Effect Size ^a^ (95% CI)	Adjusted *p* Value ^a^	Adjusted OR ^a^ (95% CI)	Adjusted *p* Value ^a^	Adjusted OR ^a^ (95% CI)	Adjusted *p* Value ^a^
**TALIA**								
T0	2.81 (−0.68, 6.30)	0.115	0.01 (−0.00, 0.02)	0.099	0.98 (0.95, 1.01)	0.276	1.02 (1.00, 1.03)	0.040
T1	1.05 (−2.44, 4.55)	0.555	0.00 (−0.01, 0.01)	0.357	0.98 (0.95, 1.02)	0.325	1.00 (0.98, 1.01)	0.789
**Sedentary-Intensity**								
T0	−15.92 (−37.41, 5.6)	0.146	0.00 (−0.05, 0.05)	0.998	1.16 (0.99, 1.35)	0.051	1.02 (0.93, 1.12)	0.661
T1	−20.62 (−38.79, −2.45)	0.026	0.00 (−0.05, 0.04)	0.883	1.14 (0.99, 1.32)	0.070	0.98 (0.90, 1.07)	0.717
**Light-Intensity**								
T0	1.17 (−4.72, 7.06)	0.697	0.00 (−0.01, 0.02)	0.589	0.98 (0.93, 1.03)	0.430	1.03 (1.00, 1.06)	0.032
T1	0.77 (−4.55, 6.08)	0.777	0.00 (−0.01, 0.02)	0.732	0.97 (0.92, 1.02)	0.267	1.00 (0.98, 1.03)	0.757
**Moderate-Intensity**								
T0	3.98 (−0.77, 8.73)	0.100	0.01 (−0.00, 0.02)	0.087	0.98 (0.94, 1.02)	0.330	1.01 (0.99, 1.03)	0.262
T1	3.32 (−2.87, 9.52)	0.293	0.01 (−0.01, 0.03)	0.228	0.98 (0.92, 1.04)	0.533	0.99 (0.96, 1.02)	0.540
**Vigorous-Intensity**								
T0	6.00 (−56.81, 68.81)	0.852	0.02 (−0.14, 0.17)	0.852	1.06 (0.65, 1.71)	0.822	0.91 (0.68, 1.21)	0.518
T1	−38.10 (−131.22, 55.03)	0.423	−0.11 (−0.35, 0.13)	0.350	1.71 (0.94, 3.11)	0.081	0.79 (0.49, 1.28)	0.333

Estimated is the effect of 10 MET-h/week change in intensities on infant anthropometrics and obstetric outcomes. ^a^ adjusted for pre-pregnancy age, pre-pregnancy BMI, parity, and group assignment. Abbreviations: BMI: body mass index. OR: odds ratio. T0: assessment before or in the 12th week of gestation. T1: Assessment after the 29th week of gestation. TALIA: Total physical activity of light intensity and above.

**Table 4:** Associations between physical activity intensity and neonatal outcomes.

	Low Birth Weight		High Birth Weight		LGA		SGA	
	Adjusted OR ^a^ (95% CI)	Adjusted *p* Value ^a^	Adjusted OR ^a^ (95% CI)	Adjusted *p* Value ^a^	Adjusted OR ^a^ (95% CI)	Adjusted *p* Value ^a^	Adjusted OR ^a^ (95% CI)	Adjusted *p* Value ^a^
**TALIA**								
T0	0.98 (0.94, 1.01)	0.181	1.02 (0.99, 1.04)	0.194	1.02 (0.99, 1.04)	0.202	0.98 (0.96, 1.01)	0.148
T1	1.00 (0.96, 1.04)	0.969	1.01 (0.98, 1.03)	0.516	1.01 (0.98, 1.03)	0.740	1.00 (0.97, 1.03)	0.937
**Sedentary-Intensity**								
T0	1.27 (1.08, 1.48)	0.004	0.93 (0.79, 1.10)	0.387	0.92 (0.77, 1.09)	0.330	0.90 (0.76, 1.06)	0.198
T1	1.25 (1.07, 1.46)	0.005	0.91 (0.78, 1.05)	0.194	0.88 (0.75, 1.03)	0.121	1.00 (0.88, 1.15)	0.979
**Light-Intensity**								
T0	0.97 (0.92, 1.03)	0.298	1.00 (0.96, 1.04)	0.895	1.00 (0.96, 1.05)	0.995	0.96 (0.92, 1.00)	0.053
T1	0.99 (0.93, 1.05)	0.711	0.99 (0.95, 1.03)	0.681	0.99 (0.95, 1.04)	0.722	0.98 (0.94, 1.02)	0.404
**Moderate-Intensity**								
T0	0.98 (0.93, 1.03)	0.403	1.03 (1.00, 1.06)	0.080	1.02 (0.99, 1.06)	0.135	1.00 (0.96, 1.03)	0.860
T1	1.01 (0.94, 1.07)	0.842	1.04 (1.00, 1.08)	0.050	1.03 (0.99, 1.07)	0.185	1.02 (0.98, 1.07)	0.383
**Vigorous-Intensity**								
T0	0.84 (0.45, 1.57)	0.579	1.08 (0.70, 1.66)	0.743	1.14 (0.72, 1.80)	0.587	1.08 (0.72, 1.63)	0.703
T1	0.97 (0.36, 2.57)	0.946	1.38 (0.77, 2.49)	0.278	1.24 (0.64, 2.40)	0.533	1.48 (0.86, 2.55)	0.160

Estimated is the effect of 10 MET-h/week change in intensities on neonatal outcomes. ^a^ adjusted for pre-pregnancy age, pre-pregnancy BMI, parity, and group assignment. Abbreviations: LGA: large for gestational age; OR: odds ratio; SGA: small for gestational age, T0: assessment before or in the 12th week of gestation, T1: assessment after the 29th week of gestation, TALIA: total physical activity of light intensity and above.

## Supplementary Materials

The following are available online at https://www.mdpi.com/2077-0383/8/10/1735/s1. Table S1: Unadjusted differences between active and inactive women in infant anthropometrics, neonatal, and obstetric outcomes. Table S2: Differences between active^T0+T1^ and inactive^T0+T1^ women in infant anthropometrics, neonatal, and obstetric outcomes. Table S3: Associations between physical activity intensity and infant anthropometrics and obstetric outcomes (unadjusted data). Table S4: Associations between physical activity intensity and neonatal outcomes (unadjusted data).

## List of Abbreviations

ACOG	American College of Obstetrics and Gynecology
BMI	Body mass index
C	Control group
CI	Confidence interval
GeliS	“Gesund leben in der Schwangerschaft”/“Healthy living in pregnancy“
IV	Intervention group
MET	Metabolic equivalent of task
OR	Odds ratio
PA	Physical activity
PPAQ	Pregnancy Physical Activity Questionnaire
RCT	Randomized-controlled trial
SD	Standard deviation
TALIA	Total physical activity of light intensity and above
LGA	Large for gestational age
SGA	Small for gestational age
