# Supplementary material for: Associations between Prenatal Physical Activity and Neonatal and Obstetric Outcomes—A Secondary Analysis of the Cluster-Randomized GeliS Trial
Source: J Clin Med. 2019 Oct 19;8(10):1735. doi: 10.3390/jcm8101735 (PMC6832262; doi:10.3390/jcm8101735)
Supplement: Supplementary file 1 [file jcm-08-01735-s001.pdf]

Table S1. Unadjusted differences between active and inactive women in infant anthropometrics, neonatal and obstetric outcomes.

	Time Point	Active		Inactive		Effect Size (95% CI)	*p* Value
		n	Mean ± SD	*n*	Mean ± SD		
**Anthropometrics**							
**Birth Weight, g**	T0	*n* = 893	3338.0 ± 527.9	*n* = 1008	3337.4 ± 508.9	0.58 (−46.04, 47.21)	0.980
	T1	*n* = 1061	3364.5 ± 481.0	*n* = 827	3341.4 ± 492.5	23.09 (−21.08, 67.26)	0.306
**Birth Length, cm**	T0	*n* = 885	51.3 ± 2.6	*n* = 1000	51.4 ± 2.6	−0.05 (−0.28, 0.18)	0.674
	T1	*n* = 1056	51.4 ± 2.4	*n* = 824	51.3 ± 2.6	0.12 (−0.11, 0.35)	0.294
**Head Circumference, cm**	T0	*n* = 875	34.7 ± 1.6	*n* = 989	34.7 ± 1.6	−0.04 (−0.19, 0.11)	0.588
	T1	*n* = 1047	34.8 ± 1.5	*n* = 814	34.7 ± 1.6	0.05 (−0.09, 0.20)	0.452
**BMI, kg/m^2**	T0	*n* = 885	12.7 ± 1.3	*n* = 1000	12.6 ± 1.2	0.02 (−0.10, 0.14)	0.737
	T1	*n* = 1056	12.7 ± 1.3	*n* = 824	12.7 ± 1.2	0.03 (−0.08, 0.15)	0.583
**BMI-z-score** [a]	T0	*n* = 884	0.05 ± 1.07	*n* = 1000	0.03 ± 0.98	0.02 (−0.07, 0.11)	0.683
	T1	*n* = 1055	0.07 ± 1.04	*n* = 824	0.04 ± 0.96	0.03 (−0.06, 0.12)	0.557
**Neonatal and obstetric outcomes**			***n* (%)**		***n* (%)**	**OR (95% CI)**	
**LGA**	T0	*n* = 893	74 (8.3)	*n* = 1006	66 (6.6)	1.29 (0.91, 1.82)	0.152
	T1	*n* = 1061	87 (8.2)	*n* = 826	54 (6.5)	1.28 (0.90, 1.82)	0.174
**SGA**	T0	*n* = 893	72 (8.1)	*n* = 1006	92 (9.1)	0.87 (0.63, 1.20)	0.402
	T1	*n* = 1061	100 (9.4)	*n* = 826	61 (7.4)	1.31 (0.94, 1.82)	0.116
**Low Birth Weight**	T0	*n* = 893	49 (5.5)	*n* = 1008	46 (4.6)	1.21 (0.80, 1.84)	0.357
	T1	*n* = 1061	46 (4.3)	*n* = 827	34 (4.1)	1.06 (0.67, 1.66)	0.810
**High Birth Weight**	T0	*n* = 893	81 (9.1)	*n* = 1008	80 (7.9)	1.16 (0.84, 1.60)	0.376
	T1	*n* = 1061	92 (8.7)	*n* = 827	66 (8.0)	1.10 (0.79, 1.52)	0.591
**Macrosomia**	T0	*n* = 893	10 (1.1)	*n* = 1008	8 (0.8)	1.42 (0.56, 3.60)	0.466
	T1	*n* = 1061	11 (1.0)	*n* = 827	7 (0.8)	1.23 (0.47, 3.18)	0.673
**Preterm Birth**	T0	*n* = 893	61 (6.8)	*n* = 1006	62 (6.2)	1.12 (0.78, 1.61)	0.555
	T1	*n* = 1061	52 (4.9)	*n* = 826	57 (6.9)	0.70 (0.47, 1.02)	0.066
**Caesarean Section**	T0	*n* = 893	264 (29.6)	*n* = 1008	278 (27.6)	1.10 (0.90, 1.35)	0.339
	T1	*n* = 1060	301 (28.4)	*n* = 827	232 (28.1)	1.02 (0.83, 1.25)	0.870

Depicted are mean ± SD and proportions (n (%)).[a] BMI-z-score was calculated using German standards [24]. *Active*: Women meeting physical activity recommendations defined as ≥ 7.5 MET-h/week in category sports activity of moderate-intensity or greater. *Inactive*: Women not meeting physical activity recommendations (< 7.5 MET-h/week in category sports activity of moderate-intensity or greater). Abbreviations: BMI: body mass index; LGA: large for gestational age; OR: odds ratio; SGA: small for gestational age; T0: assessment before or in the 12th week of gestation; T1: assessment after the 29th week of gestation.

Table S2. Differences between active^{T0+T1} and inactive^{T0+T1} women in infant anthropometrics, neonatal and obstetric outcomes.

	**Active^{T0+T1}**		**Inactive^{T0+T1}**		**Effect Size (95% CI)**	***p* Value**	**Adjusted Effect Size [a] (95% CI)**	**Adjusted *p* Value [a]**
	n	**Mean ± SD**	***n***	**Mean ± SD**				
**Anthropometrics**								
**Birth Weight, g**	*n* = 597	3357.3 ± 501.1	*n* = 1207	3353.0 ± 481.4	4.25 (−43.57, 52.08)	0.862	23.18 (−24.73, 71.10)	0.343
**Birth Length, cm**	*n* = 594	51.3 ± 2.5	*n* = 1202	51.4 ± 2.5	−0.05 (−0.29, 0.20)	0.711	0.04 (−0.21, 0.29)	0.774
**Head Circumference, cm**	*n* = 589	34.7 ± 1.5	*n* = 1188	34.7 ± 1.6	0.00 (−0.15, 0.16)	0.964	0.04 (−0.12, 0.19)	0.660
**BMI, kg/m²**	*n* = 594	12.7 ± 1.4	*n* = 1202	12.7 ± 1.2	0.03 (−0.09, 0.16)	0.591	0.07 (−0.05, 0.19)	0.271
**BMI-z-score[b]**	*n* = 593	0.08 ± 1.09	*n* = 1202	0.05 ± 0.97	0.03 (−0.07, 0.13)	0.531	0.06 (−0.04, 0.16)	0.240
**Neonatal and Obstetric Outcomes**		***n* (%)**		***n* (%)**	**Effect Size (95% CI)**	***p* Value**	**Adjusted OR [a] (95% CI)**	**Adjusted *p* Value [a]**
**LGA**	*n* = 597	54 (9.0)	*n* = 1206	81 (6.7)	1.38 (0.96, 1.98)	0.078	1.52 (1.05, 2.20)	0.025
**SGA**	*n* = 597	55 (9.2)	*n* = 1206	101 (8.4)	1.11 (0.79, 1.57)	0.552	1.03 (0.73, 1.47)	0.850
**Low Birth Weight**	*n* = 597	28 (4.7)	*n* = 1207	49 (4.1)	1.16 (0.72, 1.87)	0.533	1.07 (0.66, 1.73)	0.794
**High Birth Weight**	*n* = 597	58 (9.7)	*n* = 1207	94 (7.8)	1.27 (0.90, 1.80)	0.166	1.35 (0.95, 1.91)	0.095
**Macrosomia**	*n* = 597	8 (1.3)	*n* = 1207	10 (0.8)	1.63 (0.64, 4.14)	0.308	1.55 (0.60, 4.00)	0.371
**Preterm Birth**	*n* = 597	32 (5.4)	*n* = 1206	72 (6.0)	0.89 (0.58, 1.37)	0.601	0.86 (0.56, 1.32)	0.485
**Caesarean Section**	*n* = 597	172 (28.8)	*n* = 1207	336 (27.8)	1.05 (0.84, 1.30)	0.665	1.04 (0.83, 1.29)	0.761

Depicted are mean ± SD and proportions (n (%)).[a] adjusted for pre-pregnancy age, pre-pregnancy BMI, parity, group assignment. [b] BMI-z-score was calculated using German standards [24]. Active^{T0+T1}: Women meeting physical activity recommendations (≥ 7.5 MET-h/week in category sports activity of moderate-intensity or greater) at T0 and T1. Inctive^{T0+T1}: Women meeting physical activity recommendations at any or one time point. Abbreviations: BMI: body mass index; LGA: large for gestational age; OR: odds ratio; SGA: small for gestational age; T0: assessment before of in the 12th week of gestation; T1: assessment after the 29th week of gestation.

Table S3. Associations between physical activity intensity and infant anthropometrics and obstetric outcomes (unadjusted data).

	Birth Weight		BMI		Preterm Birth		Caesarean Section	
	Effect Size (95% CI)	*p* Value	Effect Size (95% CI)	*p* Value	OR (95% CI)	*p* Value	OR (95% CI)	*p* Value
**TALIA**								
T0	4.75 (1.37, 8.13)	0.006	0.01 (0.00, 0.02)	0.009	1.03 (1.00, 1.06)	0.098	1.00 (0.98, 1.01)	0.534
T1	3.24 (−0.17, 6.65)	0.062	0.01 (0.00, 0.02)	0.033	1.03 (0.99, 1.06)	0.107	1.01 (0.99, 1.03)	0.260
**Sedentary-Intensity**								
T0	−17.81 (−38.83, 3.21)	0.097	−0.01 (−0.06, 0.05)	0.837	0.87 (0.75, 1.00)	0.052	0.96 (0.88, 1.05)	0.340
T1	−24.73 (−42.45, −7.02)	0.006	−0.02 (−0.07, 0.03)	0.421	0.87 (0.76, 1.00)	0.050	0.99 (0.91, 1.07)	0.721
**Light-Intensity**								
T0	3.17 (-2.68, 9.01)	0.289	0.01 (−0.01, 0.02)	0.262	1.03 (0.98, 1.08)	0.273	0.98 (0.96, 1.01)	0.183
T1	2.83 (−2.42, 8.09)	0.291	0.01 (−0.01, 0.02)	0.252	1.04 (0.99, 1.09)	0.115	1.01 (0.98, 1.03)	0.651
**Moderate-Intensity**								
T0	6.63 (2.01, 11.25)	0.005	0.02 (0.00, 0.03)	0.008	1.04 (0.99, 1.08)	0.117	1.00 (0.98, 1.02)	0.876
T1	7.09 (1.04, 13.13)	0.022	0.02 (0.00, 0.03)	0.017	1.03 (0.97, 1.10)	0.293	1.02 (0.99, 1.05)	0.149
**Vigorous-Intensity**								
T0	−8.69 (−71.65, 54.27)	0.787	−0.03 (−0.18, 0.13)	0.758	0.93 (0.58, 1.48)	0.751	1.10 (0.83, 1.45)	0.504
T1	−56.72 (−150.20, 36.77)	0.234	−0.15 (−0.39, 0.09)	0.215	0.60 (0.33, 1.09)	0.093	1.32 (0.82, 2.12)	0.253

Estimated is the effect of 10 MET-h/week change in intensities on infant anthropometrics and obstetric outcomes. Abbreviations: BMI: body mass index; OR: odds ratio; T0: assessment before or in the 12th week of gestation; T1: assessment after the 29th week of gestation; TALIA: total physical activity of light intensity and above.

Table S4. Associations between physical activity intensity and neonatal outcomes (unadjusted data).

	Low Birth Weight		High Birth Weight		LGA		SGA	
	**OR (95% CI)**	***p* Value**	**OR (95% CI)**	***p* Value**	**OR (95% CI)**	***p* Value**	**OR (95% CI)**	***p* Value**
**TALIA**								
T0	1.04 (1.00, 1.07)	0.045	0.98 (0.96, 1.01)	0.119	0.98 (0.96, 1.00)	0.073	1.03 (1.00, 1.06)	0.034
T1	1.02 (0.98, 1.05)	0.421	0.99 (0.96, 1.01)	0.295	0.99 (0.96, 1.02)	0.418	1.01 (0.99, 1.04)	0.346
**Sedentary-Intensity**								
T0	0.80 (0.69, 0.94)	0.004	1.06 (0.90, 1.24)	0.484	1.08 (0.91, 1.27)	0.403	1.07 (0.92, 1.25)	0.379
T1	0.80 (0.69, 0.93)	0.003	1.10 (0.96, 1.27)	0.184	1.13 (0.97, 1.32)	0.120	0.96 (0.84, 1.09)	0.508
**Light-Intensity**								
T0	1.04 (0.99, 1.10)	0.161	1.00 (0.96, 1.04)	0.947	1.00 (0.95, 1.04)	0.838	1.05 (1.01, 1.10)	0.017
T1	1.03 (0.97, 1.09)	0.360	1.01 (0.97, 1.05)	0.779	1.00 (0.96, 1.05)	0.841	1.03 (0.99, 1.08)	0.127
**Moderate-Intensity**								
T0	1.04 (0.99, 1.09)	0.118	0.97 (0.95, 1.00)	0.040	0.97 (0.94, 1.00)	0.043	1.01 (0.98, 1.05)	0.409
T1	1.02 (0.95, 1.08)	0.663	0.95 (0.92, 0.99)	0.015	0.96 (0.92, 1.00)	0.050	1.01 (0.96, 1.05)	0.845
**Vigorous-Intensity**								
T0	1.13 (0.62, 2.06)	0.699	1.00 (0.64, 1.54)	0.982	0.98 (0.62, 1.55)	0.927	0.87 (0.59, 1.30)	0.504
T1	0.99 (0.38, 2.53)	0.977	0.77 (0.43, 1.38)	0.378	0.87 (0.45, 1.70)	0.690	0.64 (0.38, 1.09)	0.097

Estimated is the effect of 10 MET-h/week change in intensities on neonatal outcomes. Abbreviations: LGA: large for gestational age; OR: odds ratio; SGA: small for gestational age; T0: assessment before or in the 12th week of gestation; T1: assessment after the 29th week of gestation; TALIA: total physical activity of light intensity and above.
